# Biological Responses to Cadmium Stress in Liverwort *Conocephalum conicum* (Marchantiales)

**DOI:** 10.3390/ijms21186485

**Published:** 2020-09-04

**Authors:** Viviana Maresca, Gennaro Lettieri, Sergio Sorbo, Marina Piscopo, Adriana Basile

**Affiliations:** 1 Department of Biology, University of Naples “Federico II”, 80138 Naples, Italy; viviana.maresca@unina.it (V.M.); gennaroLe@outlook.com (G.L.); 2 Centro di Servizi Metrologici Avanzati (CeSMA), Microscopy Section, University of Naples “Federico II”, 80126 Naples, Italy; sersorbo@unina.it

**Keywords:** *Conocephalum conicum*, cadmium, ROS, antioxidant enzymes, *hsp* 70 gene

## Abstract

Oxidative damage (production and localization of reactive oxygen species) and related response mechanisms (activity of antioxidant enzymes), and induction of Heat Shock Protein 70 expression, have been studied in the toxi-tolerant liverwort *Conocephalum conicum* (Marchantiales) in response to cadmium stress using two concentrations (36 and 360 µM CdCl_2_). Cadmium dose-dependent production of reactive oxygen species (ROS) and related activity of antioxidant enzymes was observed. The expression level of heat shock protein (Hsp)70, instead, was higher at 36 µM CdCl_2_ in comparison with the value obtained after exposure to 360 µM CdCl_2_, suggesting a possible inhibition of the expression of this stress gene at higher cadmium exposure doses. Biological responses were related to cadmium bioaccumulation. Since *C. conicum* was able to respond to cadmium stress by modifying biological parameters, we discuss the data considering the possibility of using these biological changes as biomarkers of cadmium pollution.

## 1. Introduction

The contamination of aquatic and terrestrial ecosystems with heavy metals is an environmental problem of public health concern [1]. Cadmium (Cd) is a toxic non-essential transition metal; the third main environmental contaminant of most hazards after mercury and lead. This metal poses a health risk for both humans and animals already at very low concentrations (0.5 µM) [2,3,4,5], while for plants there is usually a wider range of concentrations (from 5 to 5000 µM) that are generally not phytotoxic to the plants [6,7]. Cd can negatively affect plant growth, and its toxic effects can be found at both morphological and physiological levels [8]. Nevertheless, the threshold of phytotoxic concentration of Cd is very different across plants and depends on species, ecotypes, cultivars, etc. [9]. Cd toxicity is well known, and the mechanisms are well understood. Effects are known at the cellular level [10]. At the molecular level, cadmium is able to bind the sulfidrilic groups of proteins and the catalytic sites of enzymes that contain oxygen, sulfur or nitrogen in the form of –OH, –COO–, –SH, –NH_2_ groups, inhibiting their biological activity or modifying their structure [11]. The effects of cadmium generally occur with an alteration of photosynthesis and respiration, mineral absorption and alterations in gene expression [12,13].

The main defense mechanism against cadmium exposure is the chelation of metals by a ligand, the phytochelatins, followed by transportation of the metal-ligand in the vacuole. This allows the metal to be subtracted from active tissues, limiting its toxic action [14,15,16,17].

Some papers indicate that bryophytes are better than lichens and leaves of vascular plants in monitoring atmospheric heavy metal pollution in urban areas, being bioindicators and bioaccumulators of metals in the environment [18,19]. More recently, numerous studies have used liverwort as bioindicators of environmental pollution [14,15,20]. In particular, the liverwort *C. conicum* has recently been used to monitor the quality of the air in the territory of Naples and, among the biomarkers considered, the increase in the presence of heat shock protein (Hsp)70 was extremely sensitive and consistent with the environmental alterations of Hsp 70 [20].

For these reasons, in this work, the liverwort *C. conicum* was used to assess the effects of exposure to two high doses of cadmium chloride which in a previous work conducted on the liverwort *Lunularia cruciata* (Marchantiales) had been found suitable for determining the activation of a whole series of defense responses [13,14,15,16,21]. In fact, in this liverwort, in response to cadmium, there is a molecular and metabolic adaptation to mitigate the toxic effects [13,15,16].

The purpose of the present work is to assess how cadmium exposure can affect *C. conicum* in order to extend this liverwort into a biomonitoring system. To this aim, after cadmium exposure we have analyzed: oxidative damage (reactive oxygen species production and localization) and related response mechanisms (activity of antioxidant enzymes such as Superoxide Dismutase (SOD), Catalase (CAT) and Glutathione S–transferase (GST)), and evaluated, by RT-qPCR, the expression of the stress genes Hsp70.

## 2. Results

### 2.1. Metal Bioaccumulation

The gametophytes of *C. conicum* grown in vitro without any cadmium treatment showed the absence of toxic ions such as antimuonium, arsenic, mercury and cadmium itself. This confirms the situation of extreme environmental preservation of the bryophyte collection site and background concentrations of metal (loid)s as Zn, Cu and Fe. Only the presence of lead was observed in traceable quantities, however it was considered negligible. After 7 days of treatment with the two solutions (36 µM and 360 µM), the concentration of cadmium in the liverwort tissues reached 162 mM and 2371 mg g^−1^ dw, respectively (Table 1).

### 2.2. Confocal Microscopy

**Figure 1:**
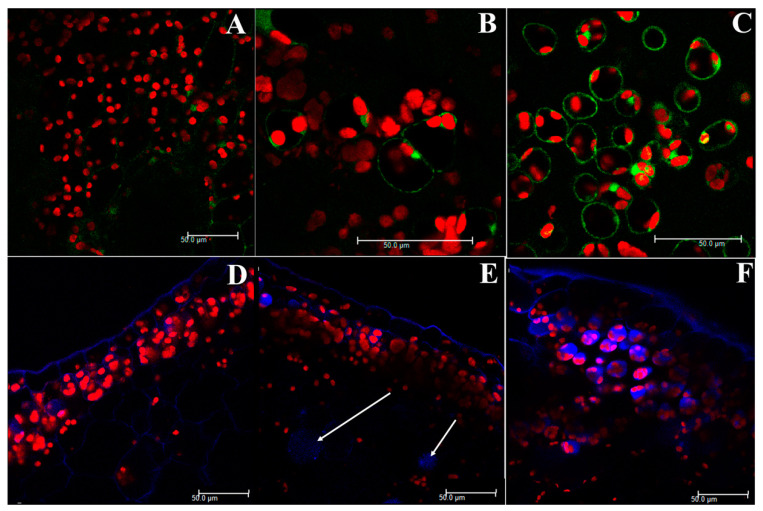
Upper thalli of *C. conicum* samples observed under the confocal laser microscope, after dichlorofluorescein (DFC)-labelling (Figure 1A–C) and monochlorobimane (MCB)-labelling (Figure 1D–F). (**A**) *C. conicum* sample from the control culture, with a faint green light and a clear red light emission from chloroplasts. (**B**) *C. conicum* sample from the 36-µM Cd culture, with a visible green light emission from the cytoplasm. The red light from chloroplasts is comparable to control. (**C**) *C. conicum* sample from 360 µM Cd culture, with clear green and red light signals. Green light is collected from the cytoplasm, red light labels the chloroplasts. (**D**) *C. conicum* sample from control culture, with a blue light from the upper epidermis cell walls and a clear red signal from the chloroplasts. (**E**) *C. conicum* sample from the 36-µM Cd culture, with a blue light from the upper epidermis cell walls and a clear red signal from the chloroplasts. Cell vacuoles emit faint blue light (arrows). (**F**) *C. conicum* sample from the 360 µM Cd culture, with a strong blue signal from cell vacuoles, blue light from the upper epidermis cell walls and a clear red signal from the chloroplasts. Scale bars: 50 µm.

Confocal microscopy examinations of DCF-labelled samples showed green signals from reactive oxygen species (ROS) in the cytoplasm and red light signal from autofluorescent chlorophyll. The green signal is barely visible in the control samples, appears faint in the 36 µM Cd-treated samples and is well visible in the cytoplasm of the 360 µM Cd-treated plants (Figure 1A–C). Red signals are comparable in all the samples. 

Confocal microscopy examinations of MCB-labelled samples showed blue and red light signals (Figure 1D–F). The blue light signal was collected from both the cell protoplasts and also the autofluorescent upper epidermis cell walls. The blue signal from protoplasts was almost absent in the control samples, barely visible in the 36 µM Cd-treated samples and maximum in the 360 µM Cd-exposed ones. The red light from chloroplasts was well visible in all the samples.

### 2.3. ROS and Antioxidant Activity Enzyme

**Figure 2:**
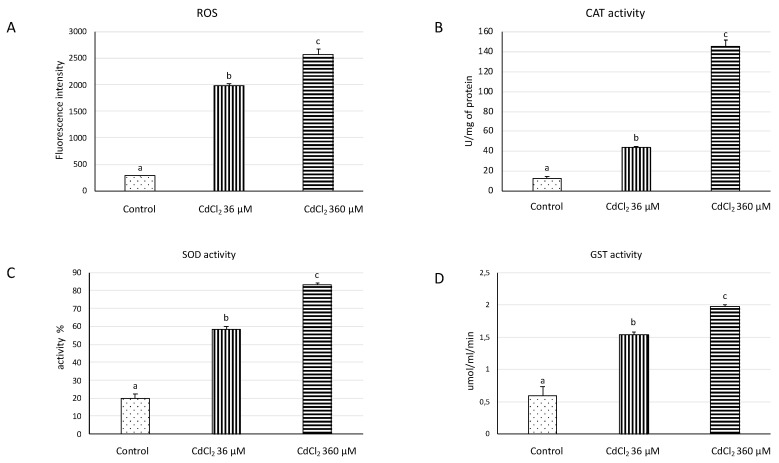
Reactive oxygen species (ROS) amount and antioxidant/detoxifying enzyme activities in *C. conicum* treated with 0 (control), 36 or 360 µM CdCl_2_ for 7 days. (**A**) ROS production; (**B**) Catalase (CAT); (**C**) Superoxide Dismutase (SOD); (**D**) Glutathione S–transferase (GST). Values are presented as mean ± st. err; (a, b, c): bars not accompanied by the same letter are significantly different at *p* < 0.05.

In Cd-treated samples, the amount of ROS highly increased compared to controls (Figure 2A), and the antioxidant/detoxifying enzymes under investigation were also progressively activated by the two Cd concentrations. Both ROS amount and enzyme activity are strongly related to its bioaccumulation (Table S1). Actually, CAT increased up to 145 U/mg for 360 µM-treated samples (Figure 2B), whereas, the activity of SOD in 360 µM-treated samples of cadmium is about 63% higher than the control, while in those treated with 36 µM there is an increase of about 25% compared to the control (Figure 2C). Both Cd concentrations markedly enhanced the GST activity, reaching a value of 2 µmol mL^−1^ min^−1^ in the 360 µM-treated samples (Figure 2D).

### 2.4. Expression of Hsp70

**Figure 3:**
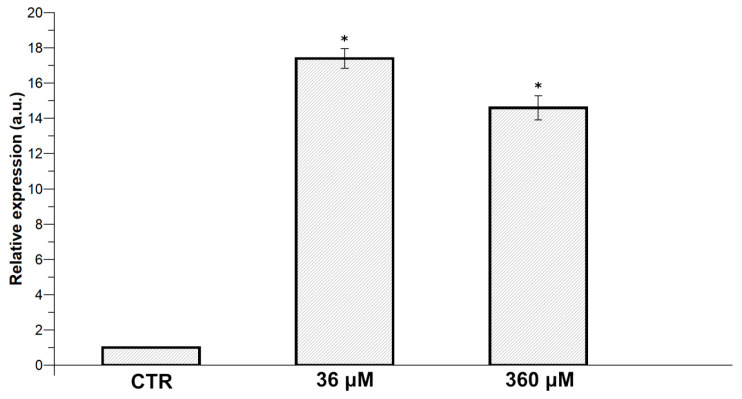
qRT-PCR expression analysis of heat shock protein (Hsp)70 gene of *C. conicum* samples exposed to 36 and 360 µM CdCl_2_. The relative expression of Hsp70 indicated as arbitrary units (a.u.) means the change in expression of the transcripts of Hsp70 genes in comparison to that of the reference housekeeping actin gene in samples exposed to CdCl_2_ with respect to unexposed samples used as control (CTR). Asterisk (*) indicates a statistically significant difference (*p* < 0.05) compared to control.

To evaluate the possible stress in *C. conicum* following to CdCl_2_ exposure, we analyzed, by RT-qPCR, the relative gene expression changes of the stress gene Hsp70, comparing the values obtained in unexposed and exposed samples. The results show an up-regulation of this gene in exposed samples since we observed an increase in the expression of Hsp70 for both exposure doses (36 and 360 µM CdCl_2_). In particular, as shown in Figure 3, the exposure to 36 µM and 360 µM CdCl_2_ produced increased Hsp70 expression ~17.4- and ~14.6-fold, respectively, relative to control condition (unexposed samples). 

## 3. Discussion

Environmental pollution is one of the major challenges in the modern human society and contamination of aquatic and terrestrial ecosystems with toxic heavy metals represents an environmental problem of public health concern [22]. Metals may accumulate in the food chain [23]; and produce loss of biodiversity and damage many species, humans included [24,25]. Biomonitoring is generally used for evaluating the effects of toxic contaminants (e.g., heavy metals) in specific areas. Organisms capable of responding to the different changes in the environment through quantifiable alterations are often employed as early warning systems to monitor such areas. Bryophyta represent very useful organisms to assess metal accumulation and have been proposed as very effective and sensitive bioindicators [26].

In our study, the liverwort *C. conicum* respond sensitively and coherently to Cd stress.

The confocal microscopy observations of DFC-labelled samples showed green and red signals, corresponding to DFC-ROS conjugates and autofluorescent chlorophyll emissions, respectively. ROS-conjugates signals were emitted from the cytoplasm, the most metabolically active compartment of the cell, while the red light came from chloroplasts. After 2′-7′dichlorofluorescein diacetate (DFC-DA) has entered the cell, it undergoes modification into DCF, which is trapped in the cytoplasm and, after reaction with ROS, can be detected as fluorescent DCF-derived compound [27]. Therefore, DCF-DA has been employed to detect intracellular oxidants in plant cells [28,29]. Our confocal microscopy observations are consistent with our chemical data showing ROS production, green signals being the highest after the 360 µM Cd treatment. Therefore, both our chemical and microscopic data show that ROS production is dose-dependent. On the other hand, ROS, even at a low concentration, are present also in the control untreated samples. That is consistent with the opinion that ROS are not only the effect of abiotic and biotic stresses, but they are also beneficial to plants, supporting cellular proliferation, physiological function, and viability, and the maintenance of basal levels of ROS in the cell is essential for life [30]. In addition, it is well known that cadmium perturbs the redox balance in plants since can produce ROS accumulation including the superoxide anion, hydroxyl radicals and hydrogen peroxide [31]. To evaluate the oxidative pressure due to Cd and its oxidative effects, we measured both ROS generation and antioxidant enzymes response. The obtained results underlined that Cd could generate ROS that lead to the development of a series of defense mechanisms such as the increase in SOD, CAT, and GST activity. In fact, in our samples, both the ROS content and the activity of antioxidant enzymes are strongly correlated to Cd bioaccumulation indicating that these enzymes are activated by the two Cd concentrations (Table S1). Thus, we can speculate that *C. conicum* owns an enzymatic arsenal that is collectively able to quench ROS even after 7 days of severe metal exposure.

Confocal observations of MCB-labelled samples showed a blue light signal, captured from both the MCB–thiol conjugates and autofluorescent upper epidermis cell walls, and red light from chlorophyll marking chloroplasts. The found blue signal from the cell protoplast was consistent with comparable findings in Cd-treated *L. riparium* after MBC labelling, where blue signal from cytoplasm and vacuoles was related to phytochelatins and other thiol groups inducted by Cd [17]. So, our findings of blue light emission from Cd-treated samples suggest noteworthy induction of thiol peptide compounds, such as γ-glutamylcysteine (γ-EC), GSH, and phytochelatins, known to be effective in mitigating harmful effect of heavy metal treatments [17].

Furthermore, comparable red signals in all treated and control samples show a well-evident chlorophyll presence, even in the highest concentration-treated samples, suggesting a preserved photosynthesis.

Several research groups have investigated how organisms respond to environmental changes to adapt to their surroundings and avoid cellular damages. Many of them evaluated the effects of these environmental changes on gene expression, protein synthesis and cell phenotype [32]. In fact, in addition to conventional markers used for toxicity testing and risk assessment in the environment, several biochemical biomarkers seem to be particularly promising, because all organisms respond to stress at the cellular level, with a rapid synthesis of the so-called stress proteins and for plants the induction of heat shock proteins (Hsps), particularly Hsp70 may be one such biomarker [33,34,35]. Hsp70 is the most conserved protein in evolution [36,37] and a highly appreciated phylogenetic nominator in the field of molecular evolution. It is present in all organisms from archaebacteria and plants to humans, and the prokaryotic protein Hsp70 DnaK shares about 50% of the identity of amino acids with the eukaryotic proteins Hsp70. Many papers report its involvement in plant temperature stress [38] and its roles in other development [39].

Hsps are mainly involved in several mechanisms of tolerance, adaptation and/or resistance to stress in living organisms [40,41], and heavy metals increased Hsp70 levels, avoiding a mis-folding of both existing proteins and newly synthetized polypeptides [42]. Data reported here show that Cd is a strong inducer of *hsp70* genes in *C. conicum*. In fact, our results clearly demonstrate that CdCl_2_ exposure increased Hsp70 expression probably to counteract the possible negative effects caused by the presence of this metal in the experimental conditions tested. We observed that *hsp*70 expression increased by about 17.4-fold after exposure of *C. conicum* to 36 µM CdCl_2_ relative to control condition (unexposed samples). Our results are in agreement with those reported in the literature for other plants for which it is reported that uptake of heavy metals enhances chaperone synthesis and occurrence, as in *Elodea canadensis* [43], *Lemna minor* [44], the moss *Leptodyctium riparium* [45,46], and the liverworts *C. conicum* [20], *Pellia neesiana* [47], and *L. cruciata* [14]. These results would suggest that this organism could be used as bioindicator of Cd environmental pollution and that hsp70 expression could be used as efficient biomarkers of Cd pollution. Our results, intriguingly, showed that the levels of Hsp70 expression were higher at 36 µM than 360 µM CdCl_2_ exposure, suggesting a possible inhibition of the expression of this stress gene at higher exposure doses. Higher concentrations of CdCl_2_ resulted in a reduction in the response, which was probably due to cytotoxicity [48], as reported for Hsp70 protein in *Fucus serratus* and *L. minor* [35].

Moreover, our results are in agreement with those obtained previously in *L. minor* after treatment with cadmium. This metal stimulated the biosynthesis of the cytoplasmic Hsp70 protein in a concentration-dependent way, higher in fronds exposed to lower doses of stressors. The results obtained by these researchers suggest that Hsp70 induction is a sensitive and nonspecific indicator of cellular stress, and may serve as an adaptive function in plants exposed mainly to low doses of cadmium [49].

The relative “resistance” of this liverwort is explained from the set of biochemical responses considered. In particular, Hsp response could protect the liverwort from proteotoxic stress just as the response of antioxidant enzymes could protect it from oxidative damage. The observed strong responses of *C. conicum* could clarify its hard resistance to pollutants and suggest it as an excellent bioindicator of pollution in the particular environments in which *C. conicum* lives, such as the margin of the rivers too often little considered in biomonitoring projects. In conclusion, for the first time to our knowledge, a study has described the effects of high doses of cadmium in *C. conicum* in vitro, by comparing both biochemical and physiological parameters such as *hsp70s*, antioxidant enzyme activity. Hsp70 levels may decrease upon exposure to higher amount to this heavy metal, due to toxic effects on metabolism but further studies are necessary to elucidate, at a molecular level, the possible relationship between the beginning of the stress and the first line of response, probably involving Hsp and other stress-related proteins.

## 4. Materials and Methods

### 4.1. Plant Material

Field-grown *Conocephalum conicum* (L.) Dum. was collected from Riccia countryside (Campobasso, Molise, Italy 41°28′50.6″ N 14°49′21.0″ E).

In particular, the liverwort *C. conicum* was randomly collected in a hilly area far from known sources of pollution and of which we had previously verified the absence of heavy metal pollution both with soil analysis and with the use of biomarkers in another liverwort [15]. The collection points should satisfy the following characteristics: *C. conicum* present on at least 20-cm-deep soil and with a cover of at least 10 cm^2^ and at least 2 km away from any road. Samples were collected from moist soil and were maintained in Petri dishes and processed in the laboratory within 6 h from collection, using the protocol of European moss survey [50]. The protocol proposed was taken into account and followed with few modifications since this was specifically developed for monitoring spatial and temporal trends in the accumulation of heavy metals in mosses at a wide scale. Specifically, samples were collected using gloves and bags in small open spaces to preclude significant effects of canopy drip. We could not follow it closely, as it considers monitoring moss on a large scale. Three pools of *C. conicum* were collected, and for each pool 6 replicates were made, for a total of 18 samples.

Field-collected specimens were utilized for in vitro cultures according the following protocol.

### 4.2. Gametophyte Culture

*C. conicum* specimens for in vitro culture were obtained cutting off the basal part (1.7–2 cm width and 3–3.2 cm of length) and transferred in laboratory controlled conditions (see after) on the same day. Single gametophytes were thoroughly washed with deionized water and then the surface sterilized for 2 min in 70% ethanol and for 2 min in 2% NaClO, with the addition of a few drops (20 µL) of Triton X-100. Subsequently, they were washed for 10 min with distilled sterile water and put on Petri dishes (5 cm diameter), 5 specimens per dish, on 20 g of fine granular, washed quartz. The specimens were cultured with 10 mL distilled sterile modified Mohr medium, pH 7.5 (100 mg KNO_3_, 10 mg CaCl_2_4H_2_O, 10 mg MgSO_4_, 136 mg KH_2_PO_4_, 0.4 mg FeSO_4_, 1 mL of Bold basal medium solution to a final volume of 1000 mL in distilled water). The heavy metals stress was induced by the supply of 36 and 360 µM CdCl_2_ for 7 days of exposition.

Control samples were supplied with NaCl at the same concentrations as heavy metal solutions. The solutions were replaced every two days across the duration of the experiments. The cultures were kept in a controlled room at 13/20 °C (night/day), 70% constant relative humidity, and 16-h light (40 mEm^−2^ s^−1^)/8-h dark photoperiod.

Gametophytes were observed every two days in order to establish the effect of metal exposure on thallus growth and browning of tissues.

### 4.3. Metal Bioaccumulation

After gathering (Riccia countryside) and after in vitro culture (36 and 360 µM), *C. conicum* gametophytes (125 mg) were dried at 105 °C for 24 h, and homogenized in an agate mortar according to Basile et al. [14]. Homogenized samples were mineralized in a microwave oven (Milestone MLS 1200 Mega, Sorisole, Italy) with 6 mL of 65% HNO_3_, 2 mL of 39% H_2_O_2_, and 0.2 mL of hydrofluoric acid (HF). The digested material was diluted in distilled water and analyzed by inductively coupled plasma-mass spectrometry (Perkin Elmer Elan 600, Norwalk, CT, USA) for Cd content. Metal contents were assayed in triplicate and measurements were repeated three times; concentrations were expressed on a dry-weight basis. Analytical quality was checked by analyzing the Standard Reference Materials ICRM 482 (*Pseudevernia furfuracea*) and CTA-VTL-2 (tobacco leaves). Precision of analysis was estimated by the coefficient of variation of five replicates and was found to be within 10% Cd content.

### 4.4. Confocal Microscopy

Fluorescent probe 2′-7′dichlorofluorescein diacetate (DCF-DA) (Sigma-Aldrich Co., St Louis, MO, USA) was used to localize intracellular ROS. The reagent, solubilized in dimethyl sulfoxide DMSO, was diluted by adding 10 mM Tris-HCl (pH 7.4), obtaining a 25 µM solution.

Thin cross sections from fresh sample thalli were incubated in the DCF-DA solution for 30 min and then washed for 10 min in the same buffer three times. The plant sections were examined under a laser-scanning confocal microscope (Leica TCS SP5, Wetzlar, Germany). Excitation sources at 405 and 496 nm were employed; the emission bandwidths were 506/585 nm (green light) and 615/715 nm (red light) (beginning–end). Objectives, the channel settings of pinhole, detector gain, amplification offset and gain were adjusted to provide an optimal balance with fluorescence intensity. Data collection and processing were carried out with the software LAS AF (Leica). Observations were repeated 3 times for control and each plant treatment.

Localization of GSH and thiol peptides was carried out labelling the samples with the fluorescent probe monochlorobimane (MCB) (Thermo Fisher Scientific, Waltham, MA, USA). MCB stock solutions were prepared at a 50-mM concentration in methanol, with subsequent dilution up to 100 μM by adding sterile water. Thin sections from fresh plant thalli, prepared as before, were incubated with 100 μM MCB (Thermo Fisher Scientific, Waltham, MA, USA) solution for 30 min at 21 °C in the dark, at near-neutral pH conditions, then washed in sterile water and observed with the same laser-scanning confocal microscope as before. An excitation source at 405 nm was employed; the emission bandwidths were 415/522 nm (blue light) and 562/741 nm (red light) (begin–end). Objectives, the channel settings of pinhole, detector gain, amplification offset and gain were adjusted to provide an optimal balance with fluorescence intensity. Data collection and processing were carried out with the software LAS AF (Leica). Observations were repeated 3 times for control and each plant treatment.

### 4.5. Detection of ROS

A fluorescent technique using 2′,7′-dichlorofluorescin diacetate (DCF-DA) has been used for quantitative measurement of ROS production. ROS quantity was evaluated according to Maresca et al. [51]. The cells were transferred to a 96-well plate, incubated with 5 μM DCF-DA for 30 min at 37 ± 1 °C and analyzed using an automatic plate reader. ROS quantity was monitored by fluorescence (excitation wavelength of 350 nm and emission wavelength of 600 nm).

### 4.6. Antioxidant Activity Enzyme

Enzyme extraction and the determination of SOD, CAT and GST activities was performed as reported in Maresca et al. [51] with some modifications. One gram (fresh weight) of plant material was ground with 1 mL of chilled NaH_2_PO_4_/Na_2_HPO_4_ buffer (PBS, 50 mM, pH 7.8) containing 0.1 mM ethylenediaminetetraacetic acid (EDTA) and 1% polyvinylpyrrolidone (PVP). The homogenate was centrifuged at 12,000× *g* for 30 min, and the supernatant (enzyme extraction) was collected for protein assay and the determination of SOD, CAT, and GST.

CAT activities were calculated and expressed as decrease in absorbance at 240 nm due to H_2_O_2_ consumption using a commercial kit (219265, Sigma- Aldrich Co., St Louis, MO, USA) and according to the manufacturer’s protocol. SOD (19160, Sigma- Aldrich Co., St Louis, MO, USA) activity was spectrophotometrically determined at 450 nm with a commercial kit (19160, Sigma-Aldrich Co., St Louis, MO, USA). GST (EC 2.5.1.18) activity was measured using a commercial kit (CS0410, Sigma- Aldrich Co., St Louis, MO, USA).

### 4.7. Total RNA Extraction, cDNA Synthesis, and Real-Time qPCR of Hsp70 Expression

Total RNA was extracted from 200 mg of leaves of unexposed and exposed to 36 µM and 360 µM CdCl_2_ *C. conicum* samples, following the procedure reported in Piscopo et al. [52]. In brief, RNA was purified using the Trizol reagent (Invitrogen, Carlsbad, CA, USA) according to the manufacturer’s instructions and then genomic DNA was removed from the samples with an Ambion DNA-free Kit (Foster City, CA, USA). cDNA was synthesized starting from 1 µg of total RNA using the M-MLV Promega Improm II kit, according to the manufacturer’s instruction and the real-time PCR (qRT-PCR) reactions were performed as described in Lettieri et al. [53]. With few modifications. In particular, to determine the expression of the hsp70 gene the PCR reactions were carried out in a 96-well plate in a 25-μL reaction volume containing 10 ng of cDNA and 0.5 µM of each forward and reverse primers. The primers used for the reaction were: hsp70 (At3g12580)-5′-GTCGAAATCATCGCCAACG-3′ and 5′-CGACTTGATTCTTGGCAGCA-3′; and actin gene was used as internal reference: actin (At3g18780)-5′-CTCCCGCTATGTATGTCGCC-3′ and 5′- TTGGCACAGTGTGAGACACAC-3′.

All PCR reactions were performed for 40 cycles with the following specifications: denaturation at 90 °C for 30 s; annealing at 60 °C for 1 min; 72 °C for 1 min. The quantification of gene expression occurred with the SYBR Green PCR Master Mix Kit (Applied Biosystems, Foster City, CA, USA) using the 7500 Real-Time PCR (Applied Biosystem Foster City, CA, USA). The relative quantification of gene expression was carried out by the ΔΔCt method [54]. The change in expression of Hsp70 transcripts from *C. conicum* exposed to 36 and 360 µM CdCl_2_ was compared to those observed in samples unexposed. All PCR reactions were performed in triplicate.

### 4.8. Pearson’s Correlation

Pearson’s correlation index was assessed under the three conditions: control, 36 µM and 360 µM, for cadmium accumulation, Hsp70 expression, ROS levels and antioxidant activity of SOD, CAT and GST. The analysis was carried out with the Microsoft Excel 2019 software, Version 2008, build 13127.20296 (*n* = 3).

### 4.9. Statistical Analysis

Significance of differences was checked with one-way ANOVA, using the Tukey test (*p* < 0.05) for post hoc comparisons. Prior to analysis, data not matching a normal distribution (Shapiro–Wilk W test at the 95% confidence interval) were log-transformed to correct for skewed distributions.

The effect of Cd concentrations in terms of ROS production, SOD, CAT, GST activities, were examined by one-way analysis of variance (ANOVA), followed by Tukey’s multiple comparison post-hoc test. Data were analyzed using the software Statistica, version 7.0 (StatSoft, Tulsa, OK, USA).

## Figures and Tables

**Table 1:** *C. conicum* gametophytes cultured in vitro without any exogenous cadmium (Cd) treatment and exposed to 36 µM and 360 µM Cd for 7 days

	Cd	Fe	Pb	Hg	Sb	As	Cu	Zn
No treatment	n.d.	83.3 ± 2.4	6.2 ± 0.3	n.d.	n.d.	n.d.	19.2 ± 2.3	32.7 ± 2.8
36 µM Cd	162.53 ± 3.9	89.1 ± 3.9	5.8 ± 0.6	n.d.	n.d.	n.d.	23.5 ± 1.8	36.4 ± 2.5
360 µM Cd	2371 ± 19	78.8 ± 5.4	6.6 ± 0.5	n.d.	n.d.	n.d.	18.7 ± 2.3	34.2 ± 3.4

Average content of metal (loid)s in *C. conicum* gametophytes prior to exogenous Cd treatment and after 36 and 360 µM treatment for 7 days (means ± SE; *n* = 6; n.d. = not detected). Metal (loid) content (mg g^−1^ dw).

## Supplementary Materials

The following are available online at https://www.mdpi.com/1422-0067/21/18/6485/s1.

## Abbreviations

GSH	Glutathione
γ–EC	γ–glutamylcysteine
GS–bimane	Glutathione–bimane
ROS	Reactive oxygen species
SOD	Superoxide dismutase
CAT	Catalase
GST	Glutathione-*S*–transferase
MCB	Monochlorobimane
HSP70	Heat Shock Proteins 70
