# Supplementary material for: Biological Responses to Cadmium Stress in Liverwort Conocephalum conicum (Marchantiales)
Source: Int J Mol Sci. 2020 Sep 4;21(18):6485. doi: 10.3390/ijms21186485 (PMC7555243; doi:10.3390/ijms21186485)
Supplement: Supplementary file 1 [file ijms-21-06485-s001.pdf]

	Cd CTR	Cd 36 µM	Cd 360 µM	Hsp70 CTR	Hsp70 36 µM	Hsp70 360 µM	ROS CTR	ROS 36 µM	ROS 360 µM	GST CTR	GST 36 µM	GST 360 µM	CAT CTR	CAT 36 µM	CAT 360 µM	SOD CTR	SOD 36 µM	SOD 360 µM
Cd CTR	1																	
Cd 36 µM		1																
Cd 360 µM		0.314170333	1															
Hsp70 CTR		-0.536115956	0.594288632	1														
Hsp70 36 µM		-0.526240498	0.600611212	0.999823477	1													
Hsp70 360 µM		-0.508225751	0.612653395	0.999226642	0.999714016	1												
ROS CTR		-0.534052102	0.594964897	0.999817913	0.999928928	0.999388441	1											
ROS 36 µM		-0.54904711	0.584344312	0.999673428	0.999509118	0.998482123	0.99979256	1										
ROS 360 µM		-0.50726321	0.614262158	0.998928763	0.998908466	0.999235575	0.998354935	0.997444961	1									
GST CTR		-0.701320062	0.44277062	0.96552245	0.963123239	0.956584439	0.966173372	0.97105005	0.952954296	1								
GST 36 µM		-0.515814003	0.608464682	0.999549936	0.999559807	0.999616055	0.999196876	0.998506646	0.999850404	0.957351002	1							
GST 360 µM		-0.524365085	0.60245334	0.99987342	0.999878683	0.999660005	0.999695375	0.999230457	0.999457688	0.961325658	0.999876974	1						
CAT CTR		-0.604176685	0.534267523	0.990358232	0.990171379	0.987178032	0.991747494	0.993573301	0.982985751	0.988389151	0.98598408	0.988476847	1					
CAT 36 µM		-0.544429007	0.588908827	0.999371544	0.998593711	0.997730753	0.99852614	0.998635446	0.998728372	0.965184084	0.999035488	0.999127732	0.987646815	1				
CAT 360 µM		-0.52957362	0.599236394	0.998861249	0.998162609	0.997776098	0.997811915	0.997526538	0.999325454	0.958735948	0.999233802	0.998955648	0.983877016	0.999713811	1			
SOD CTR		-0.414744332	0.667779095	0.987830452	0.989561545	0.992685207	0.987857982	0.984560954	0.992982728	0.914416889	0.991730221	0.99017607	0.96190535	0.985759098	0.988458553	1		
SOD 36 µM		-0.526558096	0.60139409	0.999552895	0.999180537	0.998895327	0.998891838	0.998503838	0.999671232	0.95983414	0.99979303	0.999679471	0.986040139	0.999685226	0.999791073	0.989635863	1	
SOD 360 µM		-0.543368999	0.588614893	0.999847793	0.999732168	0.998893029	0.999910764	0.999961318	0.998023205	0.968963668	0.998944554	0.999536027	0.992580264	0.998854668	0.997954441	0.985987063	0.998886566	1

Table S1. The table shows the correlation in the three different conditions, control, Cd 36 µM and Cd 360 µM, between cadmium accumulation, Hsp70 gene expression, antioxidant enzymes and ROS level. (*n*=3)
